# Knowledge Level, Attitude, and Behaviors of Farmers in Çukurova Region regarding the Use of Pesticides

**DOI:** 10.1155/2018/6146509

**Published:** 2018-07-19

**Authors:** Dilek Öztaş, Burak Kurt, Ayşegül Koç, Muhsin Akbaba, Hüseyin İlter

**Affiliations:** ^1^Department of Public Health, Yıldırım Beyazıt University School of Medicine, Ankara, Turkey; ^2^Central Community Health Center, Kastamonu, Turkey; ^3^Department of Internal Medicine Nursing, Yıldırım Beyazıt University Faculty of Health Sciences, Ankara, Turkey; ^4^Department of Public Health, Çukurova University School of Medicine, Adana, Turkey; ^5^Public Health General Directorate of Turkey Republic, Ankara, Turkey

## Abstract

**Introduction and Purpose:**

Farmers are particularly at high risk of pesticide exposure due to added risk from occupational exposure. The aim of this study is to evaluate knowledge level of farmers in the Çukurova region of the effects of pesticides, toxic symptoms, and protective equipment as well as assessing their attitudes and practices regarding pesticides.

**Material and Method:**

A total of 420 seasonal agricultural workers in Karataş District of Adana Province, Turkey, were included in the study. The questionnaire form consisting of 3 sections was administered using face-to-face interview method.

**Results:**

The mean age of the participants was 40.2 ± 10.6 years. They were engaged in farming for a mean duration of 18.5 ± 10.6 years. All of them used pesticides, but none of them had been trained on the use of pesticides. Only 26.2% of them stored pesticides in a private depot. The farmers who took empty pesticide containers to special collection bins or centers constituted only 4.3% of them. While 84.0% of them thought that pesticides could have a negative impact on human health, 5.0% of them had experienced a medical problem and 1.0% of them were poisoned after applying pesticides.

**Conclusion:**

The results show that knowledge level of farmers of safe use of pesticides is very inadequate. This lack of knowledge adversely affects workers' quality of life as well as occupational health and safety. Appropriate training programs should be organized to increase their level of knowledge.

## 1. Introduction

Agriculture is the second largest sector in the world as a source of work force [1]. In Turkey, 20.3% of the employees work in the agricultural sector and their number is 5.8 million people of whom 3.1 million are males and 2.7 million are females [2]. In other respects, agriculture is one of the most dangerous occupations among all sectors in the world. Many agricultural workers suffer from work accidents and diseases every year [1]. All individuals are confronted with some types of pesticide exposure, but farmers are particularly at high risk of pesticide exposure due to added risk of occupational exposure [3]. One of the basic principles of public health is identification and preferential protection of the risk groups [4].

Lack of education, lack of knowledge, and unintentional application errors such as handling of pesticides carelessly can pose serious health risks to farmers [5]. Concerns about the adverse effects of pesticide on health are increasing in the developing countries, especially because of low educational level and unfavorable working conditions [6].

Farmers' knowledge level on potential hazards of pesticide is very important in preventing pesticide exposure [5]. In Oluwole and Cheke's study, it was reported that efforts for training farmers are required for proper use of pesticides [7]. Perry and Layde's study emphasizes the need for training interventions aimed at increasing the awareness on pesticide safety and health risks [8]. In the study by Kalıpcı et al., it was stated that a serious education mobilization should be initiated immediately in cooperation with various institutions for educating farmers and raising their awareness regarding the issue [9]. The first step in the development of appropriate training programs to reduce pesticide hazards is to identify the extent of the problem by investigating farmers' knowledge, attitude, and perception regarding pesticide safety [10]. In addition, identifying the factors that affect farmers' use of pesticides can be an important step in the design of policies and programs [11].

This research has focused on the farmers' knowledge level as well as attitudes towards and practices on safe pesticide use in a district located in Çukurova Region. The aim of this study is to evaluate the farmers' knowledge of the effects of pesticides, toxic symptoms, and protective equipment as well as evaluating their attitudes towards and practices regarding pesticides.

## 2. Material and Method

**Figure 1:**
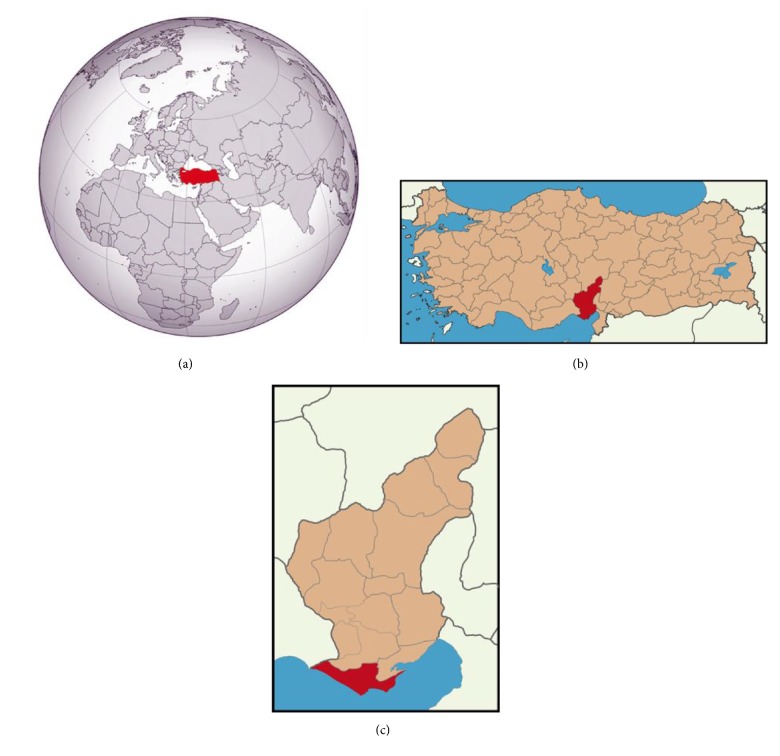
Study area: (a) Turkey, (b) Adana Province, and (c) Karataş District.

The universe of the study consisted of people who are engaged in farming in Karataş District located in the province of Adana, Turkey (Figure 1). The factors that affected the selection of this region were as follows:

- This region heavily contains agricultural labor force.
- This region has the capacity of accurately representing the Çukurova Region.
- The researchers are familiar with the region in terms of collecting reliable data.

This is a cross-sectional study. The universe of the study consists of 2500 farmers working in Karataş District of the province of Adana. No age limit was specified. In order to calculate the sample size, the expected frequency was set to 50%, the confidence interval was set to 95%, and the error margin was set to 5% on the Epi Info program. As a result, the sample size was found as 350, and it was targeted to reach 420 by adding 20% more participants to prevent losses. People were selected by simple random sampling method from the list of names obtained from the authorities.

An approval, dated 13.12.2017 and numbered 8, was obtained from the Yıldırım Beyazıt University Social and Human Sciences Ethics Committee. The interviewers were trained by the authors. Before starting the study, 40 people who worked in the same region and were not included in the sample were interviewed to run a preliminary study and the questionnaire form took its final form. After that, the questionnaire was administered with face-to-face interview method between 14.12.2017 and 04.02.2017 after the informed consent of the participants was obtained. The questionnaire is in Turkish language and was created by the researchers. It consists of three parts questioning farmers' sociodemographic characteristics, occupational characteristics, and level of knowledge about pesticides. At the end of the study, all of the 420 targeted people were reached. The data were analyzed using the SPSS 19.0 for Windows program. Frequency analysis was used.

## 3. Results

All 420 farmers included in the study were male. More than half (51.9%) were over 40 years of age; 59% had high school and above educational level (Table 1).

48.1% of the participants have been engaged in farming for more than 20 years. Among the plants they most extensively planted corn, wheat, watermelon, tomato, pepper, melon, and eggplant (Table 2).

When farmers bought pesticides, their choice was mostly based on the type of insects, effectiveness, recommendations taken from other people, and inexpensiveness. None of them had been trained about pesticides. 11.4% of them did not read warnings and precautions on the labels of pesticides (Table 3).

While 84.0% of the participants thought that agricultural pesticides could have a negative effect on human health, 5.0% had experienced medical problems after pesticide application and 1.0% were poisoned. The most common medical complaints were headache, dizziness, vomiting, and respiratory distress (Table 4).

The distribution of personal protective equipment used by the participants during spraying process is given below. Only 26.2% of the farmers kept agricultural pesticides in a private depot. Only 4.3% of the farmers took empty pesticide containers to special collection bins or centers (Table 5).

## 4. Discussion

100% of the farmers who were included in the study were male. In the study conducted by Oluwole and Cheke to determine the environmental and health effects of pesticide use in Nigeria, 93.3% of the farmers were male [7]. In the study conducted by Hashemi et al. to determine the farmers' perception of safe pesticide use, 100% of the farmers were male [6]. Similar results were obtained in our study.

The mean age of 420 farmers included in the study was 40.2 ± 10.6 years and 24.3% of the farmers were over 50 years. In the study conducted by Tuna et al. to investigate farmers' knowledge, attitudes and behaviors about pesticide storage conditions, and safe use in Kayseri/Turkey, the mean age of the farmers was found to be 51.3 ± 8.6 years [12]. Age, which is one of the socioeconomic factors, is an important factor for farmers' awareness on the prohibited and approved chemicals. Older farmers may not be aware of the use of new chemicals due to lack of knowledge [13].

Farmers' awareness of pesticides is related to their educational status. Educated farmers can read publications and access information through the Internet, thus reducing the lack of information [13]. When we examined educational status of the farmers included in the study, it was determined that 1.0% of the farmers were literate, 38.6% of them were primary school graduates, 53.3% were high school graduates, and 5.7% were university graduates. It can be said that the farmers in our study were better educated than those in other studies. In the study conducted by Yassin, Moured, and Safi on the knowledge, attitude, practice, and toxic symptoms related to the use of pesticides among Palestinian farmers, 8.5% of the farmers were uneducated, 13.2% were primary school graduates, 22.2% were secondary school graduates, 42.9% were high school graduates, and 13.2% were university graduates [14]. In the study conducted by Kalıpçı et al. to investigate educational status, level of knowledge, and environmental sensitivity of the farmers in Konya, it was reported that 55.8% of the farmers were primary school graduates, 26.6% were secondary school graduates, 11.6% were high school graduates, and 5.8% were university graduates [9].

Experience in farming is an important factor as it is an important element in acquiring skills. Experience can lead to increased production, effective input use, increased output quality and increased amount of output, and reduced costs. It is expected that experience will have a positive influence on the management ability of a farmer. The farmers who participated in our study had been engaged in farming for a mean duration of 18.5 ± 10.6 years; 15.5% of the farmers had been engaged in farming for 1-9 years, 36.4% for 10-19 years, 26.7% for 20-29 years, 13.6% for 30-39 years, and 78% for more than 40 years. In the study conducted by Ntow et al. to investigate the Ghanaian farmers' perceptions and practices with respect to pesticides, it was determined that the farmers had been engaged in farming for a mean duration of 21.2 ± 10.5 years [15].

The farmers in our study mostly raised corn (55.9%) followed by wheat (55.9%), watermelon (47.1%), tomato (38.5%), pepper (34.3%), melon (28.0%), and eggplant (25.3%) consistent with the usual practice in Çukurova region. It was determined that all of the farmers who were included in the study used pesticides this year.

When we examined the factors that the farmers who participated in our study paid attention to when purchasing pesticides, it was found that 79.5% of the farmers made a decision based on the type of insect the pesticide had an effect on, 79.3% made a decision based on the effectiveness of the pesticide, 72.4% made a decision based on recommendations, and 49.0% made a decision based on its affordability. In the study conducted by Kiraz et al., they found that when purchasing a pesticide, 52.9% of the farmers paid attention to seasonal factors, 19.0% paid attention to the quality of pesticide, and 17.3% paid attention to its effectiveness [16]. In the study conducted by Kumar et al. to determine farmers' pesticide use and their awareness on pesticides in India, it was reported that 55% of the farmers pay attention to the effectiveness of pesticides [17].

Farmers need regular training to encourage safe pesticide use and education about the risks involved in the wrong and inappropriate use of pesticides [7]. It was determined that none of the farmers who participated in our study had received any training on pesticide use. Kumar et al. reported that about 90% of the farmers had not received any training on pesticide use [17].

The label on the pesticide plays an important role in the correct use of the pesticide [18]. It was determined that 88.6% of the farmers in our study read the label/instructions on the container of the pesticide. In the study by Gaber and Abdel-Latif, 33.0% of the farmers read the instructions for using pesticides [19]. In the study by Zyoud et al., 71.4% of the farmers read the instructions [20]. Tuna et al. found that 73.0% of the farmers always read the instructions [12]. In the study conducted by Kiraz et al., 89.9% of the farmers read the label on the pesticides [16].

Farmers' knowledge of the potential damage of pesticides is very important in preventing pesticide exposure [5]. When the distribution of the farmers based on their knowledge of health effects of pesticides was evaluated, 84.0% of the farmers who participated in the study indicated that pesticides had a negative effect on human health. In the study conducted by Zyoud et al. to determine pesticide use practices and knowledge of farmers in Palestine, 85% of the farmers stated that pesticides have a detrimental effect on human health [20]. In a study conducted by Recena et al. to determine knowledge, attitudes, and practices of farmers in Brazil on pesticide exposure and pesticide use, 92.0% of the farmers stated that pesticides have a harmful effect on human health [21].

Pesticides are very harmful compounds for humans. Pesticides entering the human body can cause acute and chronic poisoning [22]. In our study, 5.0% of the farmers stated that they had a medical complaint and 1.0% of them had been poisoned due to use of pesticides. In the study conducted by Gaber and Abdel-Latif, 4.0% of the farmers stated that they had experienced poisoning [19]. In the study conducted by Ngowi, it was found that 15.0% of the farmers have been poisoned [23]. In the study conducted by Hurtig et al., it was reported that 51.8% of the farmers experienced acute poisoning [24]. It is noteworthy that the figures in our study are lower than those in the other studies. Further reasons for this should be investigated.

When the symptoms experienced by the farmers after pesticide application were examined, 3.3% of the farmers stated that they had headache, 3.3% of them had dizziness, 1.4% of them had vomiting, 1.2% of them had respiratory distress, 0.7% of them had nausea, and 0.5% of them experienced abdominal pain, diarrhea, fever, skin pruritus, and eye burning. In the study conducted by Oluwole and Cheke, 91.3% of the farmers reported that either themselves or their families experienced health symptoms associated with pesticides during or after pesticide application [7]. In the study conducted by Zyoud et al., it was reported that 37.5% of the farmers experienced itchy skin, 37.0% of them had headache, 24.9% of them experienced excessive sweating, and 21.3% of them had diarrhea [20]. In the study conducted by Yassin et al., it was reported that 64.3% of the farmers had irritation in the eyes and face, 32.4% of them had dizziness, 28.1% of them had chest pain, 27.0% of them had skin irritation, 26.5% of them had headache, 9.7% of them had abdominal pain, 8.6% of them had vomiting, 5.4% of them experienced weakness, 3.2% of them had fever, 2.7% of them has loss of libido, and 1.6% of them experienced forgetfulness [14].

Lack of personal protective equipment and unsuccessful use of pesticides are major problems during pesticide application [5]. In our study, it was determined that 64.8% of the farmers wore long sleeved shirts, 61.7% of them wore long trousers, 56.2% of them wore glasses, 51.0% of them wore gloves, 30.0% of them used masks, 27.9% of them used hats, 14.3% of them wore overalls, 6.9% of them wore boots, and 4.5% of them wore aprons. Use of personal protective equipment was also found to be low in other studies. In the study conducted by Cihan et al., 73.2% of the farmers wore gloves, 78.8% of them used masks, 15.6% of them wore boots, and 29.6% of them wore protective clothing [25]. In the study by Yassin et al., 19.6% of the farmers wore gloves, 21.7% of them used masks, 14.8% of them wore boots, and 19.0% of them wore protective clothing [14]. In a study conducted by Khan in India, it was found that only 6% of the farmers used gloves [26]. In the study by Zyoud et al., it was determined that 48.6% of the farmers wore gloves, 63.5% of them used masks, and 63% of them wore protective clothing [20]. Tuna et al. found that 37.0% of the farmers always or usually used gloves, 35.4% of the farmers always or usually used masks, and 9.5% of the farmers always or usually wore protective clothing [12]. In the study conducted by Damalas et al., it was found that 72.7% of the farmers never wore gloves, 86.8% of them never used masks, 2.5% of them never wore boots, and 81.0% of them never wore protective clothing [5]. In the study conducted by Oluwole and Cheke, it was reported that 88.9% of the farmers applied pesticides without taking any personal precautions, and only 11.1% of the farmers wore boots while preparing and applying pesticides [7].

Transport and storage of pesticide containers along with food are very dangerous. For this reason, contamination and mass influences may occur. In many countries of the world, there are strict laws preventing storage and transport of pesticides together with food. In homes, pesticides should never be stored in food or drink containers [27]. However, 78.6% of the farmers in our study stated that they kept pesticide in the general storeroom at house. In the study conducted by Yassin et al., it was stated that 18% of the farmers stored pesticides at home [14]. In the study by Hurting et al., it was found that 34.2% of the farmers stored pesticides in the house [24]. In the study conducted by Tuna et al., it was determined that 52.5% of the farmers kept pesticides in the storeroom/stock room in their houses, 17.2% kept pesticides in the kitchen, 12.7% kept pesticides in the cellar, 6.1% kept pesticides in their living space, 5.0% kept pesticides in the barn, and 5.0% kept pesticides in other areas of the house [12]. In the study conducted by Lekei and colleagues, it was found that 81% of the farmers stored pesticides in the house [28]. In the study by Oluwole and Cheke, it was found that 98.0% of the farmers stored pesticides in the house [7].

Especially in the developing countries, the use and application of pesticides under unsafe conditions and unsafe disposal of empty pesticide containers not only give harm to agricultural workers' health, but also cause serious damage to the environment and public health [29]. In our study, it was determined that 41.2% of the farmers burned empty pesticide containers, 31.0% of them threw them away, 13.6% of them buried them in the ground, 10.0% of them washed and reused them, and only 4.3% of them took them to special collection bins or centers. In the study conducted by Gaber and Abdel-Latif, 53.0% of the farmers threw away empty pesticide packages, 43.0% of them used them at home, and 4.0% of them burned them [19]. Hurting et al. found that 68.4% of the farmers threw away empty pesticide packages, 18.0% burned them, 16.2% buried them, and 38.7% of them used them to transport oil [24]. In the study conducted by Kalıpçı et al., it was found that 28.3% of the farmers buried empty pesticide packages in the ground, 23.3% of them burned them, 25.0% of them left them on the field, 14.1% of them threw them away, and 9.1% of them washed and reused them [9]. The results of our study are similar to the results of the above-mentioned studies in terms of study method. As a result of our study, it can be said that farmers are not well informed of and/or insensitive to the environmental and human health hazards of empty pesticide containers.

## 5. Conclusion and Recommendations

(1) Inadequate information on safe application of pesticides and deficiencies in the use of personal protective equipment can seriously weaken the ability of farmers to protect themselves against potential risks of pesticides. Education of farmers can be considered as one of the most important methods of eliminating unsafe use of pesticides. Education programs should target areas where farmers' knowledge is weak.

(2) The main objectives of the education that will be provided to farmers can include ensuring that they understand health hazards associated with pesticides and that they use the most appropriate protective equipment, implement personal hygiene precautions, and become aware of early symptoms of exposure.

(3) The rate of storing pesticides at home was found to be high, and this reveals that children and adolescents are at risk. In addition, it can be said that the general population is also at risk due to throwing empty pesticide containers into rubbish bin or leaving them on the field at a certain rate.

In order to protect health of people, it is necessary to make health education programs to improve attitudes and behaviors. In addition, unsafe disposal of pesticide containers by the farmers in our study indicates that in addition to solid waste collection systems it is also necessary for local administrations to work through healthy and safe collection of pesticide wastes, especially during agricultural spraying seasons.

(4) The use of protective equipment and personal hygiene habits are important in protection from pesticide exposure. In this respect, it should be ensured that farmers are provided with personal protective equipment along with pesticides and clear guidelines on protective measures must be available in the pesticide boxes, and farmers should receive health education on safe use of pesticides.

## Figures and Tables

**Table 1:** Age and education levels of participants.

		N	%
**Age**	20-29	62	14,8

	30-39	140	33,3

	40-49	116	27,6

	50-59	77	18,3

	60+	25	6,0

**Educational Level**	Illiterate	6	1,4

	Literate	4	1,0

	Primary School	162	38,6

	High School	224	53,3

	University	24	5,7

**Table 2:** Occupational characteristics of participants.

		N	%
**Duration of farming**	1-9 years	65	15,5

	10-19 years	153	36,4

	20-29 years	112	26,7

	30-39 years	57	13,6

	40+ years	33	7,8

**Planted plants**	Corn	248	59,1

	Wheat	235	56,0

	Watermelon	198	47,1

	Tomato	162	38,6

	Pepper	144	34,3

	Melon	117	28,0

	Eggplant	106	25,2

	Sunflower	32	7,7

	Peanut	21	5,0

**Table 3:** Knowledge, attitude, and behaviors of farmers about pesticides, 1.

		N	%
**Factor that is effective while purchasing pesticides**	Type of the insect	334	79,5

	Their effectiveness	333	79,3

	Recommendations	304	72,4

	Being inexpensive	206	49,0

	Availability of the pesticide in the place of purchase	20	4,8

**Status of farmers on reading warnings and precautions on labels of pesticides**	Yes	372	88,6

	No	48	11,4

**Table 4:** Knowledge, attitude, and behaviors of farmers about pesticides, 2.

		N	%
**Status of farmers on thinking that pesticides could have a negative effect on human health**	Yes	353	84,0

	No	67	16,0

**Status of farmers on having a medical problem after pesticide application**	Yes	21	5,0

	No	399	95,0

**Status of being poisoned from pesticides**	Yes	4	1,0

	No	416	99,0

**Type of complaint experienced after pesticide application**	Headache	14	3,3

	Dizziness	14	3,3

	Vomiting	6	1,4

	Respiratory distress	5	1,2

	Nausea	3	0,7

	Abdominal pain	2	0,5

	Diarrhea	2	0,5

	Fever	2	0,5

	Redness and itching on the skin	2	0,5

	Eye irritation	2	0,5

	Weakness	1	0,2

**Table 5:** Knowledge, attitude, and behaviors of farmers about pesticides, 3.

		N	%
**Personal protective equipment**	Long sleeve shirt	272	64,8

	Long trousers	259	61,7

	Glasses	236	56,2

	Gloves	214	51,0

	Protective mask	126	30,0

	Hat	117	27,9

	Overalls	60	14,3

	Boots	29	6,9

	Apron	19	4,5

**Place where pesticides are kept**	General depot	330	78,6

	Depot special to pesticides	110	26,2

	Field	53	12,6

	Barn	32	7,6

	Home garden	25	6,0

**What do farmers do with used pesticide boxes?**	They burn them	173	41,2

	They throw them away	130	30,9

	They burry them	57	13,6

	They clean and reuse them	42	10,0

	They take them to special collection bins or centers	18	4,3

## Data Availability

The data used to support the findings of this study are available from the corresponding author upon request.
